# SpeechDx: A gold‐standard speech‐and‐language dataset for prognostic AD biomarker development

**DOI:** 10.1002/alz70856_104638

**Published:** 2025-12-26

**Authors:** Lampros Kourtis

**Affiliations:** ^1^ Clinical and Translational Science Institute, Tufts University, Boston, MA, USA

## Abstract

**Background:**

Blood biomarkers are highly effective in identifying individuals with amyloid pathology, but not all patients with amyloid will go on to develop symptomatic Alzheimer's disease. Prognostic biomarkers are needed to predict who will experience future cognitive decline and are most likely to benefit from early intervention. Speech‐derived biomarkers built from acoustic and linguistic features found in speech hold significant potential as scalable prognostic biomarkers working in complement with blood biomarkers and other markers of pathology; however, their development has been hindered by the lack of large, clinically annotated speech datasets needed to train machine learning models.

**Method:**

SpeechDx is a longitudinal observational study that collects and harmonizes speech, biomarker, and clinical data from up to 3,000 participants across clinical sites in the U.S., Australia, and Spain. The SpeechDx study population was selected to capture data from participants who may experience stable cognition or cognitive decline during the 3 years of SpeechDx data collection: While participants span the full cognitive spectrum, including normal cognitive (CN), subjective cognitive decline (SCD), mild cognitive impairment (MCI) and Alzheimer's disease (AD), the majority of the study population is enrolled as CN or SCD.

Quarterly, participants remotely complete a brief battery of speech‐ and language‐eliciting tasks via a custom‐built SpeechDx app on a study‐provided tablet. Tasks are designed to elicit semi‐constrained and unconstrained speech, including picture description, story recall, storytelling, and open‐ended questions. Concurrently, clinical sites provide participant high‐quality clinical and biomarker data collection, including longitudinal blood AD biomarkers, MRI, and neuropsychological assessments. Clinical and biomarker data are paired with individual speech samples, de‐identified, and harmonized across all sites to form the unified SpeechDx Dataset. The Dataset is hosted at the AD Data Initiative Workbench, and access is managed by the Data Access Committee.

**Result:**

SpeechDx is currently enrolling participants across clinical sites in the US, Australia, and Spain, with interim data release to SpeechDx partners starting in 2025. Full dataset completion is anticipated by the end of 2028.

**Conclusion:**

SpeechDx facilitates the development of prognostic AD speech and language biomarkers through the creation of a harmonized database of longitudinal speech, biomarker, and clinical data.

